# Role of thigh circumference, calf circumference, subjective global assessment, and handgrip strength as diagnostic modalities of sarcopenia in women inflammatory bowel disease patients

**DOI:** 10.1002/jgh3.12797

**Published:** 2022-07-28

**Authors:** Rabbinu Rangga Pribadi, Marcellus Simadibrata, Andri Sanityoso Sulaiman, Murdani Abdullah

**Affiliations:** ^1^ Division of Gastroenterology, Pancreatobiliary, and Digestive Endoscopy, Department of Internal Medicine, Faculty of Medicine Universitas Indonesia Cipto Mangunkusumo National Central General Hospital Jakarta Indonesia; ^2^ Division of Hepatobiliary, Department of Internal Medicine, Faculty of Medicine Universitas Indonesia Cipto Mangunkusumo National Central General Hospital Jakarta Indonesia

**Keywords:** calf circumference, cutoff point, diagnostic accuracy, handgrip strength, inflammatory bowel disease, sarcopenia, subjective global assessment, thigh circumference

## Abstract

**Background and Aim:**

Sarcopenia is a problem affecting inflammatory bowel disease (IBD) outcome and should be evaluated by measuring muscle mass (using dual‐energy X‐ray absorptiometry [DXA]), muscle strength, and physical performance. DXA has drawbacks as it is expensive, not covered by a national program, and requires a technician. Other inexpensive and simple examinations are needed. The objective is to explore cutoff point and diagnostic accuracy of thigh circumference (TC), calf circumference (CC), subjective global assessment (SGA), and handgrip strength (HGS) to identify sarcopenia in IBD patients.

**Methods:**

The study was conducted in Cipto Mangunkusumo Hospital during November 2020–June 2021. Analysis was performed to discover the cutoff point and diagnostic accuracy of TC, CC, SGA, and HGS to identify sarcopenia.

**Results:**

As assessed by DXA, 7 of 60 women (11.7%) with IBD had sarcopenia. Using CC cutoff ≤31 cm, the sensitivity, specificity, positive predictive value (PPV), negative predictive value (NPV), positive likelihood ration (PLR), and negative likelihood ratio (NLR) were 100%, 60.38%, 25%, 100%, 2.52, and 0, respectively. Using TC cutoff ≤50 cm, the sensitivity, specificity, PPV, NPV, PLR, and NLR were 100%, 83.02%, 43.75%, 100%, 5.90, and 0, respectively. SGA has sensitivity, specificity, PPV, NPV, PLR, and NLR of 42.86%, 84.91%, 27.27%, 91.84%, 2.84, and 0.67, respectively. The area under curve of HGS was 33.3%.

**Conclusion:**

In this survey of Indonesian women with IBD, the frequency of sarcopenia was 11.7%. When compared with DXA, TC and CC values over 50 cm and 31 cm, respectively, were helpful to exclude the diagnosis of sarcopenia. SGA and HGS were of lesser value for the identification of a decrease in muscle mass.

## Graphical abstract

Sarcopenia is affecting inflammatory bowel disease (IBD) outcomes and should be evaluated by measuring muscle mass (using dual‐energy X‐ray absorptiometry [DXA]), muscle strength, and physical performance, but DXA has drawbacks as it is expensive, not covered by a national program, and requires a technician. Other inexpensive and simple examinations are needed, such as thigh circumference (TC), calf circumference (CC), subjective global assessment, and handgrip strength, to identify sarcopenia in women IBD patients. In this study, we found that the optimal cutoff points of TC and CC were more than 50 and 31 cm, and they are very good to exclude sarcopenia in women IBD patients.

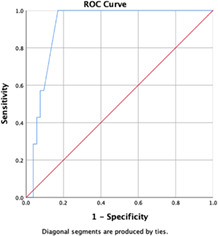

## Introduction

Inflammatory bowel disease (IBD) is a chronic bowel inflammation that consists of two main subtypes, ulcerative colitis (UC) and Crohn's disease (CD). It is related to a significant increase in mortality, morbidity, and cost.^1^, ^2^, ^3^ Bewtra *et al*.^4^ showed that all‐cause standardized mortality ratio (SMR) was 1.19 in UC and 1.38 in CD. Patients frequently have fatigue, depression, anxiety, and sleep disturbance, which affect their quality of life.^5^ The IBD cost of care was also three times higher compared with non‐IBD patients (US $ 22 987 *vs* 6956).^6^

One main issue that affects IBD outcome is sarcopenia.^7^, ^8^, ^9^ According to the Asian Working Group of Sarcopenia (AWGS), sarcopenia is decrease of muscle mass with decreased muscle strength and/or physical performance.^10^ A systematic review by Ryan *et al*.^8^ revealed that the prevalence of sarcopenia is 42% in IBD patients. Sarcopenia affects IBD outcome, as it is related to increased risk of post‐surgery complications, and faster time of biologic agent failure.^11^, ^12^

Sarcopenia has considerable impact on IBD outcome and therefore should be evaluated in all IBD patients. Sarcopenia evaluation comprises measuring muscle mass (using the gold standard dual‐energy X‐ray absorptiometry [DXA]), muscle strength (using Jamar dynamometer to measure handgrip strength [HGS]), and physical performance (using five times chair stand test).^8^ Although DXA is of utmost importance, it has several drawbacks as it is expensive and not covered by the national insurance program, and it requires a skilled technician. Those disadvantages should be addressed by evaluating other inexpensive and simple alternative examinations, for example, thigh circumference (TC), calf circumference (CC), subjective global assessment (SGA), and HGS.

The diagnostic accuracy of TC and CC had been studied by Mitayani *et al*.^13^ in geriatric population. Using women CC cutoff point of 29 cm, they revealed a 37% prevalence of sarcopenia with sensitivity, specificity, positive predictive value (PPV), negative predictive value (NPV), positive likelihood ration (PLR), negative likelihood ratio (NLR) was 71.4%, 95.5%, 62.5%, 97%, 15.87, and 0.3, respectively. Mienche *et al*.^14^ showed that by using women TC cutoff point 44 cm, the sensitivity, specificity, PPV, NPV, PLR, and NLR were 85.7%, 94%, 60%, 98.4%, 14.28, and 0.15, respectively.

SGA also has the potential to identify sarcopenia, yet its diagnostic accuracy is not known in IBD patients.^15^ In gastrointestinal patients (including IBD), Onishi *et al*.^16^ revealed that SGA has sensitivity, specificity, PPV, NPV, PLR, NLR of 19.05%, 84%, 25%, 78.75%, 1.19, and 0.96. Another tool such as HGS showed a moderate positive correlation with muscle mass in gastrointestinal cancer patients.^17^ Cutoff point and diagnostic accuracy of TC, CC, SGA, and HGS had been shown in other populations but are not fully elucidated in Indonesian women IBD populations. Therefore, the objective of this study is to explore the cutoff point and diagnostic accuracy of TC, CC, SGA, and HGS to identify sarcopenia in women Indonesian IBD patients.

## Methods

The diagnostic study was a cross‐sectional study conducted in Cipto Mangunkusumo National Central General Hospital from November 2020 until June 2021. The inclusion criteria were all IBD patients who came to the gastroenterology clinic aged 18–59 years old. The exclusion criteria were cognitive dysfunction, depression, hand deformities, and subject who refused to participate.

The examination of the subjects includes demographic characteristic, clinical characteristic, muscles mass/lean mass using DXA, muscle strength/HGS using Jamar dynamometer, physical performance using five times chair stand test (5CST), CC, TC, and SGA. Sarcopenia was diagnosed using the AWGS 2019 criteria: appendicular skeletal muscle index (ASMI) less than 7 kg/m^2^ for men and less than 5.4 kg/m^2^ for women accompanied with HGS less than 26 kgf for men and less than 18 kgf for women or 5CST (men and women) equal or more than 12 s.^10^

Statistical analysis was performed to find optimal cutoff points and diagnostic accuracy of CC, TC, and SGA to identify sarcopenia in IBD patients and HGS to identify decrease of muscle mass in IBD patients. Diagnostic accuracy analysis generates sensitivity, specificity, PPV, NPV, PLR, and NLR. This study was approved by a local institutional review board.

## Results

A total of 85 subjects participated, and the median age was 42 years (min‐max: 18–58 years old). There were 29.4% male and 70.6% female subjects. The proportion of sarcopenia was 11.67% in 60 female subjects and 12.9% in all 85 subjects. The proportion of UC and CD was 56.5 and 43.5%, respectively, among all subjects.

**Figure 1:**
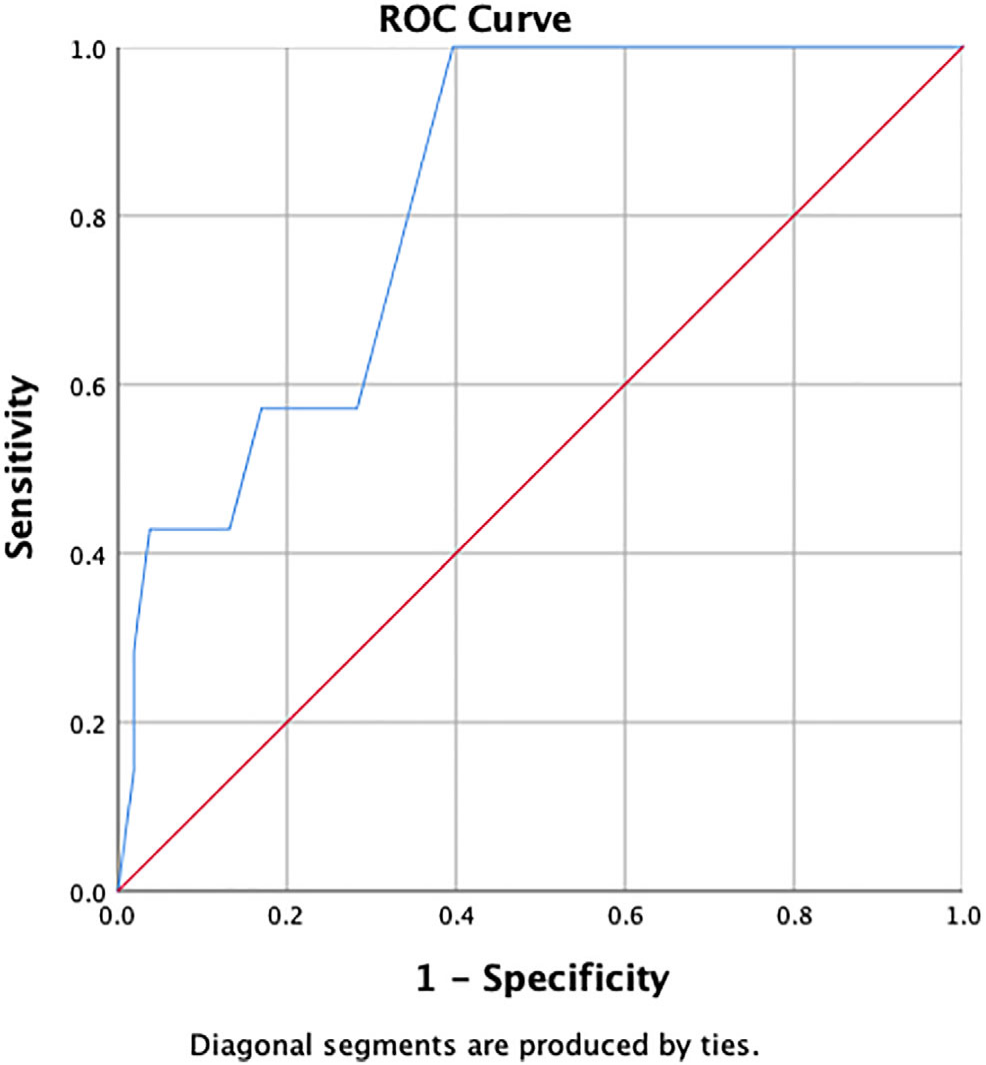
Receiver‐operating characteristic (ROC) curve of calf circumference in identifying sarcopenia for women inflammatory bowel disease patients

In women IBD patients, the area under curve (AUC) of CC was 82.5% (95% confidence interval [CI] 69.2–5.7) with *P* value = 0.006. The optimal cutoff point of CC was 31 cm, and it generates sensitivity, specificity, PPV, NPV, PLR, and NLR of 100%, 60.38%, 25%, 100%, 2.52, and 0. The AUC of CC is depicted in Figure 1.

**Figure 2:**
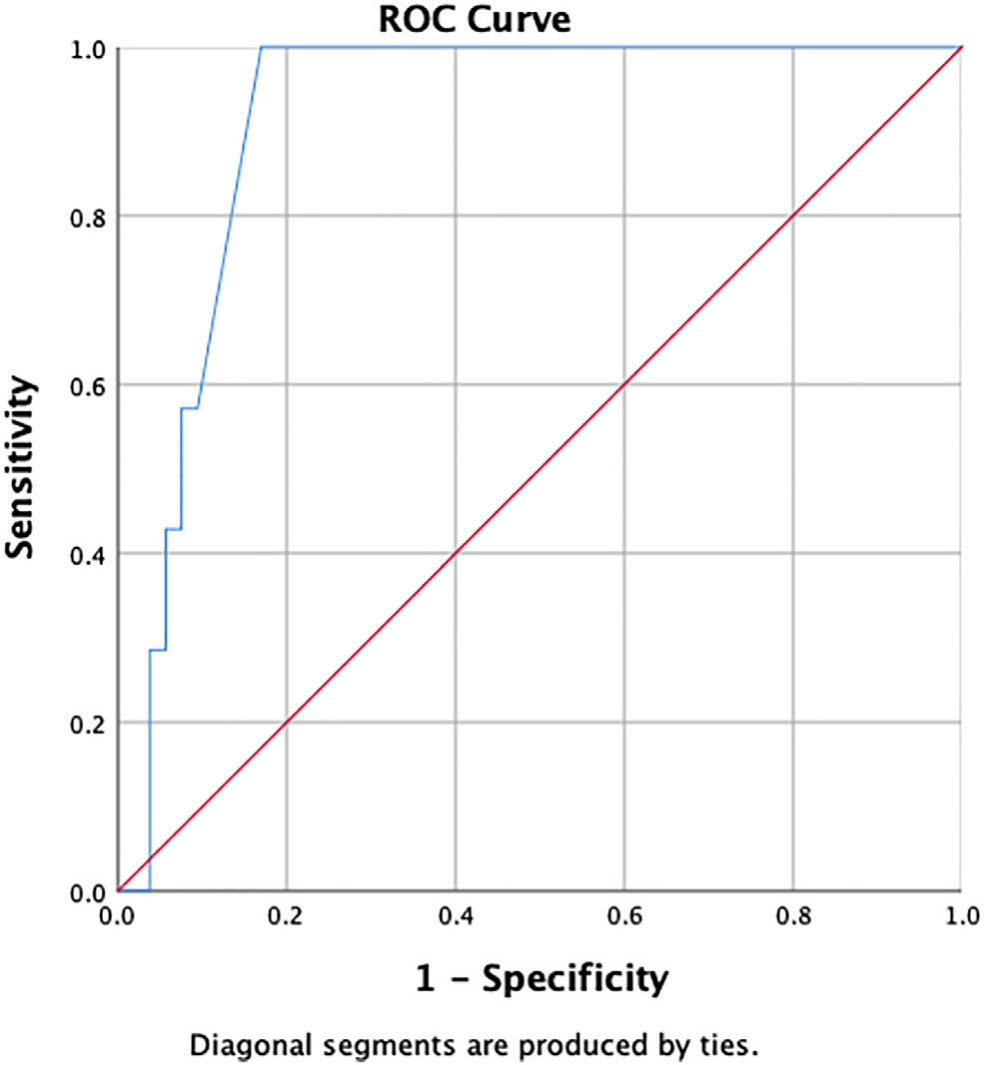
Receiver‐operating characteristic (ROC) curve of thigh circumference in identifying sarcopenia for women inflammatory bowel disease patients

In women IBD patient, the AUC of TC was 91.4% (95% CI 84.2–98.6%) with *P* value < 0.001. The optimal cutoff point of TC was 50 cm. From the current cutoff point, it generates sensitivity, specificity, PPV, NPV, PLR, and NLR of 100%, 83.02%, 43.75%, 100%, 5.89, and 0. The AUC of TC is depicted in Figure 2.

In women IBD patient, the diagnostic accuracy of SGA to identify sarcopenia is as follows: sensitivity is 42.86%; specificity is 84.91%; PPV is 27.27%; NPV is 91.84%; PLR is 2.84; and NLR is 0.67. The AUC of HGS to identify decrease of muscle mass was 33.3% (95% CI 18.9–47.8), *P* = 0.067 in women IBD patient group. Cutoff points and diagnostic accuracy of HGS were not explored further.

## Discussion

Our study used AWGS 2019 criteria as the reference standard in diagnosing sarcopenia in IBD patients. The criteria are usually applied to geriatric population. It is not generally used in IBD patients; however, it is currently the best diagnostic criterion for sarcopenia in Asian population.

The AUC of CC is 82.5% (95% CI 69.2–95.7), *P* value = 0.006. If CC is performed on women IBD patients in clinical practice, it will measure precisely in 82 of 100 patients. If CC is measured repeatedly in 100 patients in population, the correct value will be obtained in 69–95 patients. In a group of women IBD patients, the AUC of TC is 91.4% (95% CI 84.2–98.6), *P* value < 0.001. If TC is measured repeatedly in 100 patients in population, the correct value will be obtained in 84–98 patients. Statistically, the AUC of TC is more accurate compared with CC.

Cutoff point for CC and TC was 31 and 50 cm. Using CC cutoff ≤31 cm, the sensitivity, specificity, PPV, NPV, PLR, NLR were 100%, 60.38%, 25%, 100%, 2.52, and 0. Using TC cutoff ≤50 cm, the sensitivity, specificity, PPV, NPV, PLR, and NLR were 100%, 83.02%, 43.75%, 100%, 5.90, and 0. Overall, the diagnostic accuracy of TC is more superior than CC. The sensitivity of both TC and CC is very good (100%), with specificity of TC is higher than CC (83.02 *vs* 60.38%). The NPV and NLR of both TC and CC are very good (100% and 0), with PPV of TC being slightly higher than CC (43.75 *vs* 25%). From the comparison, it is decided that TC is the parameter that can be utilized in clinical practice to exclude sarcopenia in women IBD patients. The TC cutoff of 144 cm from previous research (Mienche *et al*.) was not used in this study because it was applied in ambulatory geriatric patients. While our subjects were IBD women patients less than 60 years old whose muscle mass and/or function differ from geriatric patients.

The sensitivity, specificity, PPV, NPV, PLR, and NLR of SGA for identifying sarcopenia in women IBD patients were 42.86%, 84.91%, 27.27%, 91.84%, 2.84, and 0.67. The specificity is good, but the sensitivity, NPV, PLR, and NLR are not accurate enough. The NPV is good for excluding sarcopenia, but SGA is not as simple as CC or TC, and therefore it is not a preferable tool to exclude sarcopenia in women IBD patients. The HGS has an AUC of 33.3% in detecting low muscle mass in women IBD patients. Due to its low AUC, HGS cutoff point and diagnostic accuracy of HGS are not analyzed.

Our study has limitations. The diagnostic accuracy and new cutoff point cannot be applied to IBD women geriatric patients. As for the external validity and generalizability, the result of the study is not yet applicable for IBD women patients outside Cipto Mangunkusumo National General Hospital due to possible difference in disease severity compared with other healthcare facilities.

In conclusion, the optimal cutoff point of TC and CC is 50 and 31 cm, respectively. The diagnostic accuracy of both TC and CC is very good to exclude sarcopenia in female IBD patients. SGA is not accurate enough to exclude nor diagnose sarcopenia in female IBD patients. HGS is not accurate enough to identify the decrease of muscle mass in female IBD patients.
